# A Facile Synthesis Strategy for N-Doped Graphene Quantum Dots Electrode Materials: Electrochemical Behaviors and Universal Energy Storage Mechanism

**DOI:** 10.3390/ma18235373

**Published:** 2025-11-28

**Authors:** Yongbo Wang, Shichao Dai, Jinghe Guo, Yanxiang Wang, Bo Tang

**Affiliations:** 1 College of Chemistry, Chemical Engineering and Materials Science, Shandong Normal University, Jinan 250014, China; 2 Key Laboratory for Liquid-Solid Structural Evolution and Processing of Materials, State Key Laboratory of Crystal Materials, Shandong University, Jinan 250061, China

**Keywords:** N-GQDs, hydrothermal reactions, electrochemical testing, ASC, DFT

## Abstract

In this paper, a simple hydrothermal approach is employed to prepare nitrogen-doped graphene quantum dots (N-GQDs) with controllable size and structural features, where citric acid and ethylenediamine served as the carbon and nitrogen precursors, respectively. The influence of hydrothermal temperature and duration on the structural features, surface chemistry, and electrochemical behavior of N-GQDs is systematically investigated. The capacitive behavior of N-GQD electrodes exhibits typical pseudocapacitive characteristics, primarily attributed to the surface functional groups. The NG-2 electrode (180 °C, 6 h) demonstrates a specific capacitance of 309.8 F g^−1^ at 1 A g^−1^ and maintains 98.1% of its initial capacitance after 8000 cycles, confirming excellent stability. Density functional theory (DFT) results demonstrate that the co-presence of graphitic and pyrrolic nitrogen induces a synergistic modulation of the electronic structure, resulting in improved charge-transfer kinetics and surface reactivity of N-GQDs compared to single-type nitrogen doping. Additionally, NG-2//activated carbon (AC)-asymmetric supercapacitor (ASC) achieves an energy density of 22.5 Wh kg^−1^ at 500 W kg^−1^ and maintains outstanding cycling stability. This work provides valuable insights into the design and application of N-GQDs for advanced energy storage devices.

## 1. Introduction

As society and the economy advance, energy consumption continues to increase steadily. However, the limited and non-renewable nature of traditional fossil fuels (coal, oil, and natural gas) has led to their gradual depletion, posing a serious threat to global energy security [1,2,3]. The development of renewable energy sources (such as wind, hydro, and solar power) is an effective measure to enhance energy security. However, their large-scale application is still restricted by regional dependence, climatic variability, and high development costs. Therefore, the development of advanced energy storage devices capable of rapid and safe energy storage is of great practical importance [4,5]. Currently, common energy storage devices mainly include batteries and capacitors. Batteries (lead-acid batteries, lithium batteries, and sodium-ion batteries) store and release energy primarily through redox reactions [6]. Due to their high energy density, they are widely used in new energy vehicles, smart devices, and electrical appliances. However, low power density, poor cycling performance, long charging times, and certain safety risks limit their application [7,8]. Capacitors are primarily divided into two categories: electrostatic capacitors and SCs. Owing to their high power density, fast charge–discharge capability, excellent cycling durability, and eco-friendly nature, SCs are extensively used in portable electronics, emergency power systems, and flexible devices [9,10]. However, the low energy density of SCs remains a critical limitation for high-performance applications [11]. Therefore, developing high-performance SCs with high energy and power density, excellent rate capability, and outstanding cycle stability remains a significant challenge.

In recent years, owing to their abundant active sites, excellent electrical conductivity, large specific surface area, and good dispersibility, N-GQDs have been extensively applied in SCs [12]. In comparison with other carbon-based materials, including activated carbon, carbon nanotubes (CNTs), and carbon aerogels [13], the surface of N-GQDs contains numerous structural defects and functional groups, which can allow N-GQDs to adsorb electrolyte ions and provide abundant active sites for electrode reactions. When N-GQDs serve as structural bridges or conductive components in the construction of composite materials (N-GQDs/CNFs [14], N-GQDs/Fe_2_O_3_ [15], and N, S-GQDs/NiCo_2_S_4_ [16]), the introduction of N-GQDs effectively optimizes the interfacial structure of the composite material and improves their electrical conductivity, thus enhancing the overall electrochemical performance of the composite electrodes. In addition, N-GQDs can effectively prevent material aggregation, thereby leading to an enlarged specific surface area and improved electrolyte ion adsorption capacity of the composite electrodes [17]. Lu et al. [18] prepared N-GQDs/Ni-Co-Se composite electrodes using a simple hydrothermal method. The highly conductive N-GQDs improved the electron or ion transport ability of the composite electrode, and the hybrid device exhibited superior energy density and outstanding long-term stability. Li et al. [19] prepared N-GQDs/cMOF-5 composite electrodes using hydrothermal and electrochemical deposition methods. Thanks to the excellent surface activity and pseudocapacitive properties of N-GQDs, the specific capacitance of the N-GQDs/cMOF-5 electrode was 780 F g^−1^, and the hybrid device exhibited good cycling stability. N-GQDs, owing to their unique structural dimensions and excellent dispersibility, can be readily integrated with other materials to construct high-performance electrode materials. Therefore, the electrochemical properties of N-GQDs have become particularly important. The size, structure, and composition of N-GQDs play a critical role in determining their surface activity and electronic structure, which in turn significantly affect their electrochemical performance. However, few studies have systematically explored these factors, leading to insufficient theoretical understanding to support the application of N-GQDs in energy storage systems. Furthermore, although nitrogen atoms can be incorporated into GQDs as graphitic, pyrrolic, and pyridinic nitrogen, the specific effects of these nitrogen configurations on the structure and properties of GQDs remain poorly understood, and the underlying energy storage mechanism of N-GQDs has yet to be fully elucidated.

Computer simulation is widely used in materials science, biomedicine, and environmental science due to its ability to calculate various properties of materials at the microscopic level (atoms, molecules), thereby revealing their reaction mechanisms, kinetic characteristics, and performance predictions [20,21,22]. Therefore, computational simulations can bridge the gaps left by experimental studies. The basic principle of DFT is based on the Hohenberg–Kohn theorem to obtain the electron density of a multi-electron system by solving the Kohn–Sham formula, thereby describing the energy of the system [23,24,25]. DFT calculations can be employed to analyze the density of states, band structure, and adsorption energy of materials, thereby revealing their electronic, optical, and thermodynamic characteristics. Guo et al. [26] utilized DFT calculations to determine the electronic structure, electrostatic potential, and formation energy of N/S co-doped GQDs, successfully demonstrating that heteroatom doping significantly enhances the electron transport properties of GQDs. Therefore, DFT simulations can be used to calculate the electrostatic potential, HOMO-LUMO charge, and electronic density of states of N-GQDs, revealing their behavior in electron transport, surface activity, and reaction kinetics. In addition, the combination of experiments and simulations can effectively verify the synergistic effect between graphite nitrogen and pyrrole nitrogen or pyridine nitrogen, and clarify their doping mechanism.

In this paper, the effects of hydrothermal conditions on the structure, surface functional groups, and electrochemical properties of N-GQDs were systematically studied through both experimental characterization and theoretical simulations. The N-GQDs electrode was optimized using electrochemical activation to achieve superior electrochemical performance, and its potential for energy storage applications was evaluated through the assembly of ASCs. The results indicated that N-GQDs synthesized under hydrothermal conditions of 180 °C for 6 h were uniformly distributed within the carbon matrix, exhibiting well-defined crystallinity and rich surface functional groups. The N-GQDs electrode delivered 309.8 F g^−1^ at 1 A g^−1^ and maintained 98.1% capacitance after 8000 cycles. DFT results confirmed the synergistic enhancement of conductivity and surface activity by graphitic and pyrrolic nitrogen. The ASC exhibited 22.5 Wh kg^−1^ at 500 W kg^−1^ with excellent cycling stability. This work offers both experimental and theoretical insights into the energy storage mechanism of N-GQDs for practical applications.

## 2. Experimental Section

### 2.1. Experimental Materials

All reagents are of analytical grade and used without further purification. Citric acid (CA) and ethylenediamine (EDA) were obtained from McLean Reagent Co., Ltd. (Shanghai, China). Potassium hydroxide (KOH), acetone, and ethanol were purchased from China National Pharmaceutical Chemical Reagent Co., Ltd. (Beijing, China). Activated carbon (AC), conductive carbon black, and polytetrafluoroethylene (PTFE, 60 wt%) solution were supplied by Fuzhou Yihuan Carbon Co., Ltd. (Fuzhou, China). Button battery cases (CR2032), cellulose membranes, and nickel foam were obtained from Cyber Electrochemical Materials Network (Luoyang, China).

### 2.2. Preparation of N-GQDs

A homogeneous mixture containing 3 g of CA and 1 g of EDA in 30 mL of deionized water was hydrothermally treated at 180 °C for 6 h. The resulting orange-yellow solution was filtered, dialyzed, and subsequently freeze-dried to yield pale-yellow N-GQDs, denoted as NG-2. The effects of hydrothermal temperature and reaction time on the structural characteristics of N-GQDs were systematically investigated by preparing a series of control samples under various conditions: 160 °C-6 h, 200 °C-6 h, 180 °C-4 h, 180 °C-8 h, and 180 °C-12 h. The resulting samples were labeled NG-1, NG-3, NG-4, NG-5, NG-6, respectively. The experimental parameters are listed in Table S1.

### 2.3. Preparation of N-GQDs Electrode

Nickel foam was cleaned with acetone, ethanol, 0.1 M HCl, and water, dried at 60 °C, and cut into 1 × 1 cm electrodes. A slurry of NG-2, carbon black, and PTFE (8:1:1) in ethanol was sonicated for 30 min and evenly applied onto the foam. The foam nickel loaded with active substances was pressed into electrode sheets at 5 MPa after vacuum drying at 60 °C for 12 h, with the NG-2 mass of 2 mg.

### 2.4. Electrochemical Activation of the NG-X Electrode

Cyclic voltammetry (CV) activation was used to activate the NG-2 electrode with a voltage window of −0.6–0.6 V at 100 mV s^−1^ for 200 cycles.

### 2.5. Assembly of NG-2//AC-ASC

The ASC, composed of NG-2 and AC as the positive and negative electrodes, was labeled NG-2//AC. According to the charge balance principle, the mass ratio between the positive and negative electrode materials was calculated using the Formula (1):

(1) $$\frac{m_{+}}{m_{-}}=\frac{C_{-}\;\times {∆V}_{-}}{C_{+}\times {∆V}_{+}}\;$$

Here, *m*, *C*, and ∆*V* represent the mass of the active material, specific capacitance, and potential window, respectively. The total mass of active substances in ASC is 5 mg.

### 2.6. Material Characterization

The morphology and crystal structure of N-GQDs were characterized using transmission electron microscopy (TEM, JEM-F200, JEOL Ltd., Tokyo, Japan) and scanning electron microscopy (SEM, SU-70, Hitachi, Tokyo, Japan). The height distribution and three-dimensional morphology of NG-2 were analyzed by atomic force microscopy (AFM, BioScope Resolve, Bruker Ltd., Billerica, MA, USA). Raman spectroscopy (Raman, PHS-3C, HORIBA, Kyoto, Japan) with a 433 nm laser source was employed to investigate the surface structure and defect characteristics of N-GQDs. The surface functional groups were identified by Fourier transform infrared spectroscopy (FTIR, Tensor II, Bruker Ltd., Berlin, Germany). X-ray photoelectron spectroscopy (XPS, AXIS SUPRA, Shimadzu, Kyoto, Japan) with a monochromatic Al Kα source (hν = 1486.6 eV) was used to analyze the elemental composition and valence states of N-GQDs. The XPS scale was calibrated with a sample work function of 4.26 eV, resulting in a uniform +0.39 eV energy shift applied to all spectra to eliminate variations from adventitious carbon referencing [18,27].

### 2.7. Electrochemical Testing

The NG-2 electrode and ASC were evaluated electrochemically using a CHI660E workstation (Shanghai Chenhua, Shanghai, China) with 6 M KOH as the electrolyte.

The NG-2 electrode was tested in a three-electrode system using platinum and a saturated calomel electrode as the counter and reference electrodes, respectively. The CV measurements were performed within a potential window of −0.6 to 0.6 V. Galvanostatic charge–discharge (GCD) tests were carried out in the same voltage range with current densities ranging from 1 to 10 A g^−1^. Electrochemical impedance spectroscopy (EIS) was conducted over a frequency range of 10^−2^ to 10^5^ Hz using an amplitude of 5 mV.

The ASC device was charged under constant current, and the connected LED operated at a rated voltage of 2.1 V.

The specific capacitance of the NG-2 electrode and ASC was calculated according to the following Formula (2):

(2) $$C\;=\;\frac{I\cdot ∆t}{m\cdot ∆V}$$

where *C* (F g^−1^), ∆*T* (s), *m* (g), and ∆*V* (V) denote the specific capacitance, discharge time, active mass, and potential window, respectively.

The energy density (E) and power density (P) of the ASC were determined according to Formulas (3) and (4), respectively:

(3) $$E\;=\;\frac{1000}{2\cdot 3600}\;C\Delta V^{2}$$

(4) $$P=\frac{E\cdot 3600}{∆t}$$

where *E*, *C*, ∆*V*, *P*, and ∆*T* represent the energy density (Wh kg^−1^), specific capacitance (F g^−1^), voltage window (V), power density (W kg^−1^), and discharge time (s), respectively.

The relationship between peak current and scan rate was fitted using Formula (5) to analyze the capacitive behavior of the NG electrode.

i = av^b^ log^i^ = blog^v^ + log^a^ (5)

where i (A) and v (V s^−1^) denote the peak current and scan rate, respectively, and a and b are fitting constants. A b-value of 0.5 corresponds to a diffusion-controlled process, whereas a b-value of 1 reflects a surface-controlled capacitive behavior.

In order to further analyze the proportion of pseudo current in the NG-2 electrode, Formula (6) is used to analyze the current and scan rate:

(6) $$I=k_{1}v+k_{2}v^{0.5}\;\frac{I}{v^{0.5}}=k_{1}v^{0.5}+k_{2}$$

where I (A) and v (V s^−1^) denote the current and scan rate, respectively, and k_1_ and k_2_ are proportional constants. The component *k*_1_*v* is associated with surface-controlled processes, while *k_2_v^0.5^* arises from diffusion-controlled charge storage.

### 2.8. Theoretical Calculation

DFT calculations for N-GQDs were carried out using the DMol^3^ module implemented in Materials Studio 2017, which employed numerical functions based on atomic-centered grids as its atomic basis set [28]. The GGA-PBE functional was applied because of its effectiveness in describing π-electron density and its accuracy in predicting the electronic and structural properties of graphene [29]. Spin-unrestricted calculations were applied to accurately describe the spin polarization and electronic distribution of N-GQDs. For geometric optimization, the self-consistent field (SCF) convergence criteria were set with an energy change threshold of 10^−6^ Ha and a maximum force threshold of 0.02 eV Å^−1^. For electronic structure calculations, the SCF convergence criteria required an energy change threshold of 10^−6^ Ha or an electron density change (Δρ) threshold of 10^−6^ a.u. 

For the construction of N-GQDs: The graphite crystal belongs to the hexagonal system with a space group of P63/MMC and lattice parameters of *a* = *b* = 2.46 Å, *c* = 6.8 Å, and angles α = β = 90°, γ = 120°. The GQDs model is derived by cleaving the graphite crystal along the (001) plane and constructing a 5 × 5 supercell along the *x* and *y* directions. To build N-GQDs, several carbon atoms are substituted with nitrogen and oxygen atoms, forming models containing pyrrolic-N, graphitic-N, and oxygen-containing functional groups. To avoid interactions between periodic replicas, a vacuum region of 15 Å was set along the *z*-axis.

## 3. Results and Discussion

**Figure 1:**
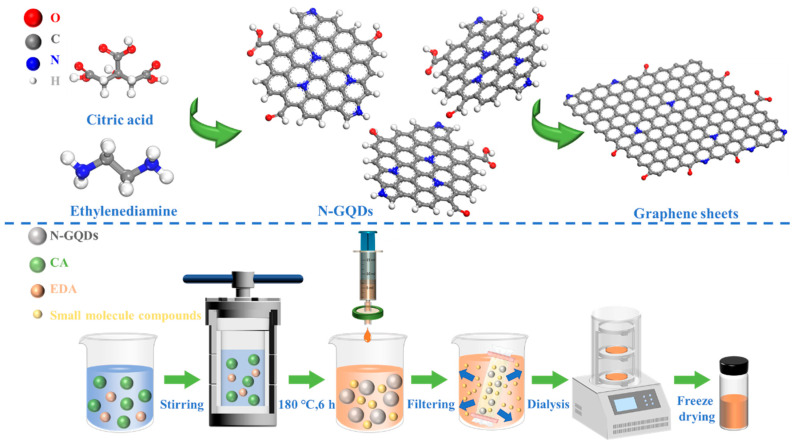
Synthesis diagram of N-GQDs.

The synthesis of N-GQDs is schematically illustrated in Figure 1. Firstly, the carboxyl and hydroxyl groups in CA undergo dehydration reactions under high temperature and pressure, resulting in the formation of aromatic alkenyl structures. These small molecular structures further undergo condensation reactions to form carbon core structures. Nitrogen atoms from EDA react with carbon sources to form nitrogen species that, as the hydrothermal reaction proceeds, embed into the carbon network as graphitic, pyrrolic, and pyridinic nitrogen. Eventually, the carbon core structure grows and develops, ultimately forming N-GQDs with the graphene structure. The structure and size of N-GQDs are closely related to the hydrothermal temperature and reaction time. The N-GQDs aggregate and grow into large-sized graphite sheets at excessively high temperatures or for an extended period of time. Furthermore, the abundant functional groups on N-GQD surfaces create multiple active sites, facilitating electrochemical processes.

### 3.1. Structural Differences of N-GQDs Synthesized Under Different Hydrothermal Conditions

**Figure 2:**
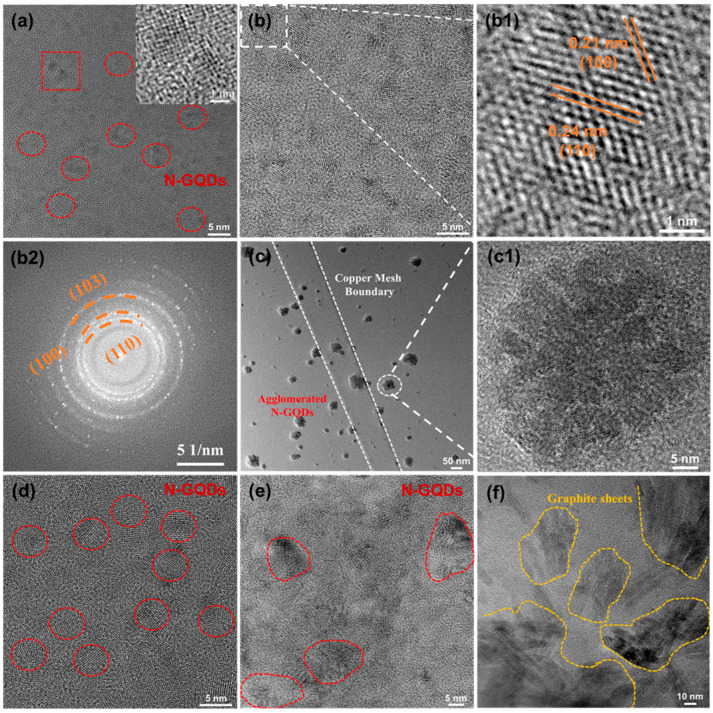
TEM images: (**a**) NG-1, the inset shows the HRTEM image of NG-1, (**b**) NG-2, (**b1**) HRTEM image of NG-2, (**b2**) SAED pattern of NG-2, (**c**,**c1**) NG-3, (**d**) NG-4, (**e**) NG-5 and (**f**) NG-6.

The size and structural evolution of N-GQDs are strongly affected by hydrothermal temperature and reaction duration. As shown in Figure S1, the color of the solution gradually deepens as the temperature or reaction time increases. Slight black precipitates were observed in the NG-3 and NG-6 solutions, which can be attributed to the formation of large graphite sheets. Notably, weak blue emission under 365 nm UV confirms successful N-GQD formation [30,31]. TEM was employed to further examine the influence of hydrothermal conditions on the morphology and structure of N-GQDs, as shown in Figure 2. NG-1 and NG-4 (Figure 2a,d) exhibit relatively small particle sizes, with average diameters of 2.37 and 2.76 nm, respectively (Figure S2). Small-sized N-GQDs display more pronounced quantum and edge effects. Under hydrothermal conditions of 180 °C for 6 h, NG-2 is uniformly dispersed within the carbon film (Figure 2b), exhibiting an average particle size of approximately 3.75 nm (Figure S3) with an elliptical morphology. The average height of NG-2 is about 2.83 nm, corresponding to roughly eight graphene layers (Figure S4). As shown in Figure 2b1, NG-2 displays well-defined graphitic lattice fringes with interplanar spacings of 0.24 nm and 0.21 nm, corresponding to the (110) and (100) planes of graphene, respectively, confirming its high crystallinity [32]. Additionally, the Selected Area Electron Diffraction (SAED) pattern of NG-2 (Figure 2b2) reveals the (100), (110), and (103) crystal planes of graphene, and the well-defined crystalline structure endows NG-2 with excellent electrical conductivity and structural stability.

However, the N-GQDs gradually undergo aggregation and growth. For NG-3, the N-GQDs still maintain an elliptical shape, but the aggregation of N-GQDs (Figure 2c) reduces the specific surface area and surface activity of N-GQDs. The average size of NG-3 is approximately 32.56 nm, and the magnified image (Figure 2c1) further confirms the aggregation of N-GQDs. Moreover, as the hydrothermal time further increases, small-sized N-GQDs tend to grow into larger graphite sheets. (Figure 2f). Large-sized N-GQDs exhibit a reduced specific surface area and longer diffusion paths for electron and ion transport. The above analysis indicates that the size and structure of N-GQDs are strongly influenced by the hydrothermal conditions.

### 3.2. Analysis of Surface Functional Groups and Elemental Valence States of N-GQDs Synthesized Under Different Hydrothermal Conditions

**Figure 3:**
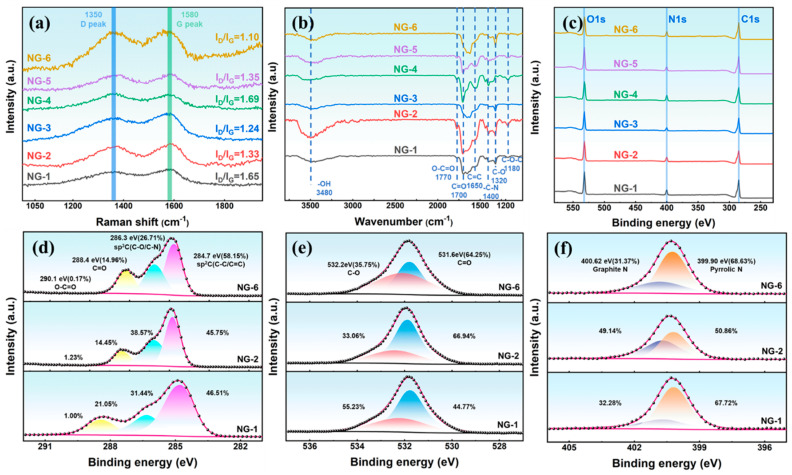
(**a**) Raman spectra, (**b**) FTIR spectra, (**c**) XPS spectra of N-GQDs; High-resolution XPS spectra of (**d**) C 1s, (**e**) O 1s, (**f**) N 1s.

Figure 3a presents the Raman spectra of N-GQDs, showing two prominent peaks at 1350 cm^−1^ (D band) and 1580 cm^−1^ (G band), which are characteristic of graphitic carbon [33]. The intensity of the D peak reflects the number of defects in graphene, while the G peak represents the ordered structure in graphene. Therefore, the value of I_D_/I_G_ is used to characterize the surface regularity of carbon materials, with higher values indicating more surface defects [34]. As shown in Figure 3a, I_D_/I_G_ gradually decreases with increasing hydrothermal time or temperature, indicating improved surface regularity of N-GQDs due to further carbonization at higher temperatures.

As shown in Figure 3b, the characteristic peak at 1650 cm^−1^ corresponds to the C=C skeletal vibration, while the peaks at 3480, 1700, 1650, and 1320 cm^−1^ are attributed to the –OH, O–C=O, C=O, and C–O groups of NG-2, respectively. In addition, the presence of the characteristic peak at 1400 cm^−1^, associated with C-N stretching, confirms successful nitrogen doping into the GQDs. To further understand the influence of hydrothermal conditions on the composition of N-GQDs, XPS is performed to investigate the changes in the valence states of elements in N-GQDs, as shown in Figure 3c–f. The XPS full spectra (Figure 3c) show that N-GQDs have three characteristic peaks at 285.32 eV, 400.29 eV, and 532.29 eV, corresponding to C 1s, N 1s, and O 1s, respectively. The XPS results (Figure 3d–f) confirm that N-GQDs are rich in oxygen- and nitrogen-containing functional groups. The C 1s spectra (Figure 3d) show four components at 285.32 eV (C–C/C=C), 286.69 eV (C–O/C–N), 288.79 eV (C=O), and 290.49 eV (O–C=O). As the hydrothermal temperature or duration increases, the sp^2^ carbon content rises, accompanied by a decrease in sp^3^ C and C=O components, which can be attributed to the rearrangement of oxygen-containing groups on the N-GQDs surface. For NG-2, the proportions of sp^3^ C and C=O are 38.57% and 14.45%, respectively, indicating abundant oxygen- and nitrogen-containing surface groups that supply plentiful electroactive sites. The O 1s spectrum (Figure 3e) shows two peaks centered at 531.99 eV (C=O) and 532.29 eV (C–O). As the hydrothermal reaction progresses, the C-O groups are gradually oxidized into C=O groups. Notably, NG-2 contains 66.94% C=O species, which can generate pseudo-capacitance via surface redox reactions [35,36]. High-resolution N1s spectra (Figure 3f) indicate that the graphite nitrogen and pyrrole nitrogen contents in NG-2 are 50.86% and 49.14%, respectively. Graphitic nitrogen improves the conductivity of N-GQDs by adjusting the electron density distribution around carbon atoms, while pyrrolic nitrogen contributes to pseudo-capacitance through redox reactions [17,19,37]. The synergistic effect of graphitic and pyrrolic nitrogen endows NG-2 with excellent conductivity and abundant active sites, significantly improving its electron transport capability and enhancing its pseudocapacitive properties.

### 3.3. Electrochemical Performance of N-GQDs Synthesized Under Different Hydrothermal Conditions

**Figure 4:**
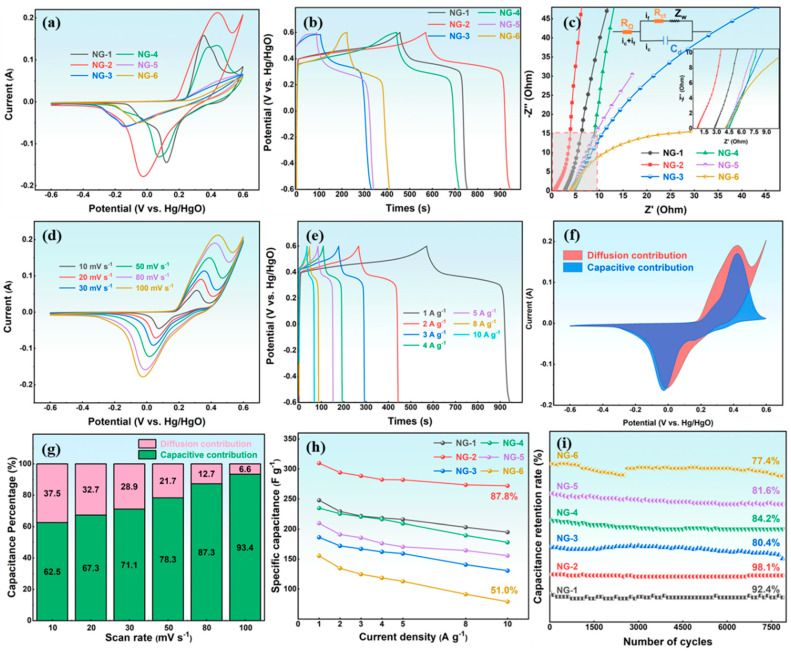
(**a**) CV curves (100 mV s^−1^), (**b**) GCD curves (1 A g^−1^), and (**c**) EIS curves of N-GQDs; (**d**) CV curves, (**e**) GCD curves, (**f**) Diffusion and capacitive contributions (80 mV s^−1^), and (**g**) Capacitive contribution ratios at different scan rates of NG-2; (**h**) The specific capacitance, and (**i**) The cyclic curves of N-GQDs.

Before electrochemical testing, the N-GQDs electrode was activated by 200 CV cycles at 100 mV s^−1^ to improve its structure and surface characteristics. Figure 4a presents the CV curves of N-GQDs at 100 mV s^−1^, showing typical pseudocapacitive behavior with distinct redox peaks at approximately 0.40 and −0.03 V. The positive and negative shifts in the oxidation and reduction peaks are attributed to reduced active sites on the electrode surface or increased impedance due to polarization [38]. Furthermore, the oxidation peaks gradually deform, indicating irreversible redox reactions that significantly impact the rate performance of the capacitance. Notably, NG-2 exhibits the largest CV area, indicating the highest specific capacitance. The GCD curves of N-GQDs (Figure 4b) display distinct charge–discharge plateaus. And the discharge time of N-GQD electrodes first increases and then decreases with changing synthesis conditions, reflecting the influence of particle size, structure, and surface chemistry. Among them, NG-2 shows the longest discharge duration, confirming its superior electrochemical performance. Additionally, all N-GQD electrodes show minimal vertical voltage drops, suggesting low internal resistance, which facilitates efficient electron and ion transport.

EIS was performed to further examine the reaction kinetics and internal resistance of the N-GQD electrodes, as shown in Figure 4c. The Nyquist plots consist of a semicircle in the high-frequency region, a 45° line at intermediate frequencies, and an almost vertical line at low frequencies [39,40]. The intercept of the semicircle on the X-axis corresponds to the equivalent series resistance (Rs), the diameter represents the charge transfer resistance (Rct), the 45° segment corresponds to Warburg diffusion, and the vertical line at low frequency signifies an ideal capacitive response [41]. It can be observed that the semicircles in the EIS curves are almost negligible, reflecting the rapid charge transfer capability of N-GQDs at high frequencies. According to Table S2, it can be seen that the R_s_ values of NG-1, NG-2, NG-3, NG-4, NG-5, and NG-6 are 2.76 Ω, 0.74 Ω, 4.12 Ω, 4.49 Ω, 4.21 Ω, and 3.98 Ω, respectively. Among them, the internal resistance of NG-2 is the smallest, indicating that NG-2 has efficient electron transfer. Furthermore, the nearly vertical line for NG-2 demonstrates its ideal capacitive behavior [42]. Ion diffusion behavior of the N-GQDs electrode was assessed by fitting Zʹ versus ω^−1/2^ in the Warburg region, where the slope of the linear fit corresponds to the diffusion resistance. According to Figure S5a, NG-2 shows the smallest slope, suggesting superior ionic diffusion properties. Figure S5b shows the frequency-dependent variation in imaginary capacitance (C″). The peak frequency defines the relaxation time constant (τ_0_ = 1/f), representing the minimum discharge time of the electrode. A smaller τ_0_ value indicates faster ion diffusion and charge-transfer kinetics, thus implying superior rate capability. The τ_0_ values of NG-2, NG-3, and NG-6 are 0.15 s, 0.45 s, and 0.47 s, respectively, revealing that NG-2 possesses a faster electrochemical response and better rate performance.

The CV curves of N-GQDs at different scan rates (Figure S6) verify their pseudocapacitive behavior. As the scan rate rises, the redox peaks shift slightly while retaining clear shapes, reflecting good rate capability. To further explore the charge-storage mechanism, the dependence of peak current on scan rate was analyzed. As shown in Figure S7, NG-2 exhibits a *b*-value of 0.55, indicating that its capacitance is mainly governed by a diffusion-controlled process. As shown in Figure 4d, NG-2 shows distinct redox peaks at different scan rates, confirming surface-controlled capacitance. The peaks shift slightly with scan rate while retaining sharp profiles, demonstrating good rate capability. The oxidation peak near 0.6 V corresponds to carboxyl group oxidation with poor reversibility, while the peak around 0.3–0.45 V arises from pyrrolic-N or hydroxyl oxidation [19,35]:


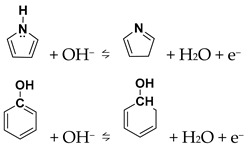


Figure 4e is the GCD curves of NG-2 at different current densities. With increasing current density from 1 A g^−1^ to 10 A g^−1^, the GCD curves exhibit minimal distortion, revealing the superior rate performance and rapid charge–discharge kinetics of NG-2. According to Formula (5), the pseudo current of the NG-2 electrode is fitted to analyze its pseudo-capacitance behavior, and the proportion of pseudo capacitance is quantitatively analyzed using the area ratio, as shown in Figure 4f,g and Figure S8. The proportion of pseudo-capacitance is 63% at 10 mV s^−1^. When the scanning speed increases to 100 mV s^−1^, the proportion of pseudo-capacitance is 93%. This reflects the efficient electron transfer ability and rapid oxidation-reduction reaction of NG-2 at high current density. As shown in Figure S9, the N-GQDs exhibit distinct charge–discharge plateaus, confirming their pseudocapacitive behavior. The curve shapes remain nearly unchanged with increasing current density (1–10 A g^−1^), indicating excellent rate performance. The specific capacitance of NG-1, NG-2, NG-3, NG-4, NG-5, and NG-6 is 247.9 F g^−1^, 309.8 F g^−1^, 186.5 F g^−1^, 234.8 F g^−1^, 209.8 F g^−1^, and 155.6 F g^−1^, respectively, as shown in Figure 4h. NG-2 maintains a capacitance of 272 F g^−1^ with 87.8% retention at 10 A g^−1^, demonstrating its superior rate performance. In contrast, NG-6 exhibits only 79 F g^−1^ and 51.0% retention, reflecting its poorer electrochemical kinetics.

The practical applicability of the N-GQD electrode was evaluated through 8000 GCD cycles at 15 A g^−1^, as illustrated in Figure 4i. The capacitance retention rates of NG-1, NG-2, NG-3, NG-4, NG-5, and NG-6 are 92.4%, 98.1%, 80.4%, 84.2%, 81.6%, and 77.4%, respectively. Among them, NG-2 exhibits the best electrochemical stability compared to other GQDs-based electrode materials, as summarized in Table S3. Moreover, the morphology of NG-2 shows no significant change after cycling, indicating its excellent structural stability (Figure S10). The above analysis demonstrates that the NG-2 electrode exhibits outstanding electrochemical performance, attributed to: (1) Its optimal size and structure maximize the accessible surface area; (2) Abundant oxygen- and nitrogen-containing surface groups supply numerous active sites for redox reactions; (3) The synergistic effect of graphitic and pyrrolic nitrogen, enhancing conductivity and contributing to pseudo-capacitance; (4) Its low internal resistance, promoting efficient charge and ion transport.

### 3.4. Evolution of Structure and Components of NG-2 During the Activation Process

**Figure 5:**
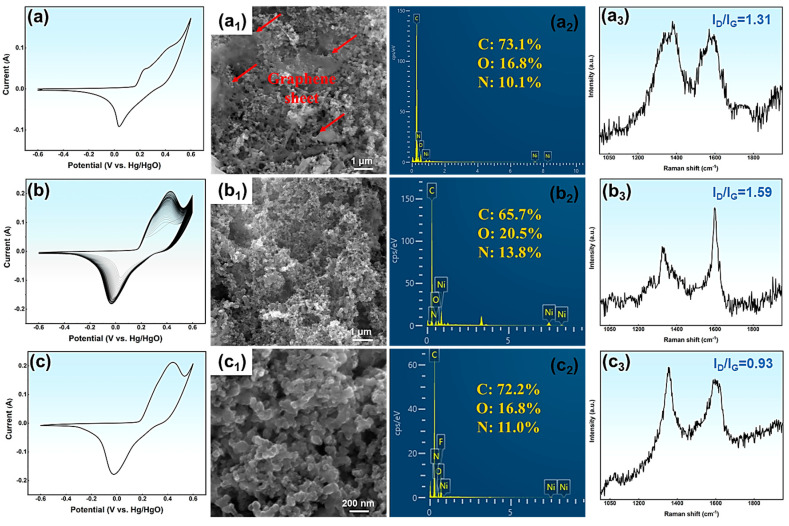
The CV curves, SEM images, EDS, and Raman spectra: (**a**–**a_3_**) ONG-2, (**b**–**b_3_**) NG-2, (**c**–**c_3_**) CNG-2.

To explore the activation mechanism and track the structural and compositional changes of N-GQDs during activation, SEM and Raman characterization are performed on the original NG-2 electrode (ONG-2), activated NG-2 electrode (NG-2), and cycled NG-2 electrode (CNG-2), as shown in Figure 5.

The CV curve of ONG-2 in Figure 5a exhibits pseudocapacitive behavior, confirming that energy storage mainly originates from surface redox processes. However, the CV area is relatively small, which fails to fully exploit the pseudocapacitive characteristics of N-GQDs. As activation progresses, the CV curve area gradually increases (Figure 5b), suggesting an enhancement in the energy storage capacity of the NG-2 electrode.

Figure 5c shows the CV curve of the CNG-2 electrode after 8000 cycles at 15 A g^−1^. The curve remains nearly identical to that of NG-2, confirming its excellent cycling stability. From the SEM images (Figure 5a_1_–c_1_), the ONG-2 electrode consists mainly of N-GQDs with some large graphite sheets, which weaken surface reactivity and decrease the number of active sites. The bonding between the graphite sheets and the substrate primarily involves van der Waals forces or electrostatic adsorption, which are relatively weak interactions. The graphite sheets are gradually oxidized and etched during activation, and finally removed from the substrate by the electric field. Consequently, the NG-2 electrode is composed of uniformly distributed N-GQDs (Figure 5b_1_) after CV activation. After 8000 cycles, the surface morphology of the CNG-2 electrode remains largely unchanged (Figure 5c_1_). EDS analysis of the NG-2 electrode further reveals the effects of CV activation on the composition of N-GQDs. During activation, the carbon content in the NG-2 electrode gradually decreases, while the oxygen and nitrogen contents increase, which enhances the pseudo-capacitance of the NG-2 electrode (Figure 5a_2_,b_2_). The main reasons for the compositional changes in NG-2 include: (1) Oxidation and detachment of graphene sheets; (2) Oxidation reactions of active carbon atoms at the edges of NG-2 with surrounding protons; (3) Further oxidation of functional groups on the NG-2 surface during CV activation. The oxygen and nitrogen contents of CNG-2 slightly decrease after 8000 cycles. Raman spectra are further used to analyze the surface order and defects of NG-2, as shown in Figure 5a_3_–c_3_. Compared with the ONG-2 electrode, the NG-2 electrode shows a slightly higher ID/IG ratio, suggesting an increase in surface defects. These variations stem from the partial breakdown of microcrystalline graphite areas, reorganization or oxidation of surface groups, and progressive etching of surface defects. The decreased ID/IG ratio of CNG-2 after cycling reflects improved surface regularity. In conclusion, the remarkable electrochemical performance of the NG-2 electrode results from several synergistic factors, including: (1) The uniform size distribution and good crystal structure of NG-2, which endows it with excellent conductivity and structural stability; (2) The abundant surface functional groups on NG-2 provide numerous electroactive sites and enhance surface reactivity, thereby reducing ion diffusion resistance; (3) These functional groups also contribute pseudo-capacitance through reversible redox reactions; (4) During CV activation, the large graphite sheets on the NG-2 surface are gradually removed, exposing more N-GQDs and increasing the electrode’s effective surface area. This structural evolution facilitates ion and electron transport, ultimately enhancing the electrochemical performance of NG-2.

### 3.5. Research on the Application of NG-2 Electrode in Asymmetric Supercapacitors

**Figure 6:**
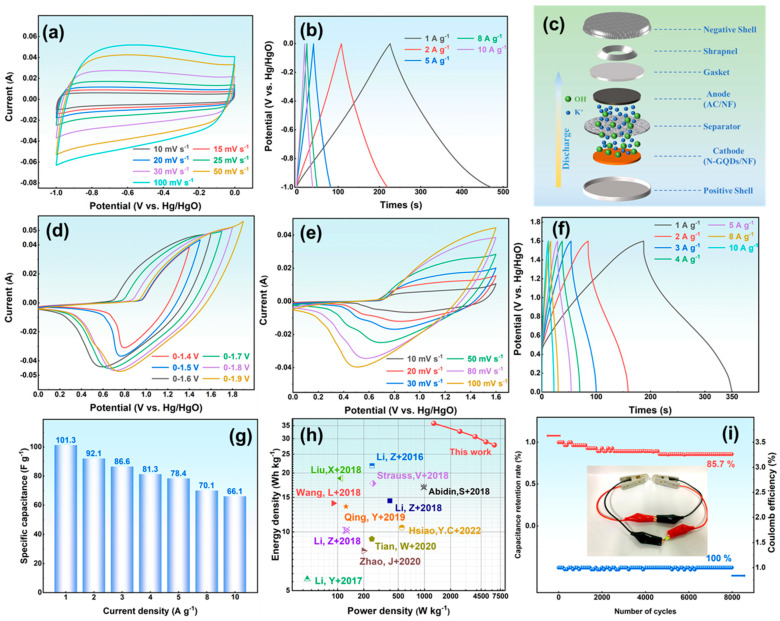
(**a**) CV curves, (**b**) GCD curves of AC; (**c**) Schematic diagram of NG-2//AC-ASC; (**d**) voltage window, (**e**) CV curves, (**f**) GCD curves, (**g**) Specific capacitance, (**h**) The energy density and power density [14,17,19,43,44,45,46,47,48,49], and (**i**) The cycle curve of ASC.

AC is used as the negative electrode to broaden the voltage window of the ASC. As shown in Figure 6a, its CV curves display a nearly rectangular shape, confirming electric double-layer capacitance. The potential range (−1.0–0 V) matches the NG-2 electrode, ensuring proper voltage balance. The GCD curves (Figure 6b) reveal capacitances of 245.5 F g^−1^ at 1 A g^−1^ and 192.4 F g^−1^ at 10 A g^−1^. The assembled NG-2//AC ASC (Figure 6c) shows ideal capacitive behavior at a 0–1.6 V window (Figure 6d), confirming efficient utilization of both electrodes. The CV curves (Figure 6e) display both double-layer and pseudocapacitive behavior. With higher scan rates, the oxidation and reduction peaks shift slightly but maintain defined shapes, reflecting excellent rate performance. The GCD curves (Figure 6f) show asymmetric triangular profiles with stable charge–discharge plateaus, and their shapes remain nearly unchanged from 1 A g^−1^ to 10 A g^−1^, demonstrating efficient ion/electron transport.

The ASC exhibits specific capacitances of 162.1 and 105.6 F g^−1^ at 1 and 10 A g^−1^, respectively, corresponding to a retention of 65.1% (Figure 6g). The Ragone plot (Figure 6h) demonstrates an energy density of 22.5 Wh kg^−1^ at 500 W kg^−1^ and 14.7 Wh kg^−1^ at 4981.3 W kg^−1^, exceeding most reported N-GQD-based supercapacitors [14,17,19,43,44,45,46,47,48,49]. After 8000 cycles at 10 A g^−1^ (Figure 6i), the ASC preserves nearly 100% Coulombic efficiency and 85.7% capacitance retention, confirming excellent durability. Two ASCs connected in series can light an LED, demonstrating good practical potential.

### 3.6. The Effect of Nitrogen Doping on the Electronic Structure and Surface Activity of N-GQDs

To further analyze the effects of nitrogen doping on the properties of N-GQDs and explore the synergistic interaction between pyrrolic nitrogen and graphitic nitrogen, DFT calculations are performed for N-GQDs. Figure S11 is the atomic models of N-GQDs with different nitrogen doping configurations. Figure S11a–d correspond to pristine GQDs, N-GQDs doped with two graphitic nitrogen, N-GQDs doped with two pyrrolic nitrogen, and N-GQDs doped with both graphitic and pyrrolic nitrogen. Their molecular formulas are C_54_H_18_, C_52_H_18_N_2_, C_56_H_19_N_2_, and C_54_H_19_N_4_, respectively, and are named GQDs, G-NG, P-NG, and GP-NG.

**Figure 7:**
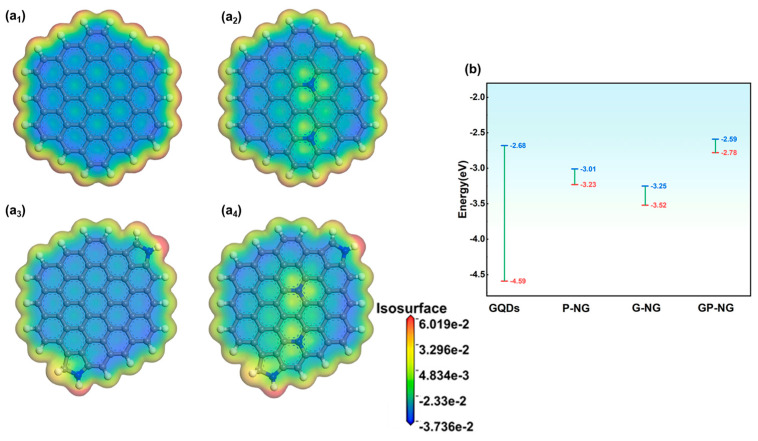
(**a_1_**–**a_4_**) Electrostatic potential and electron density of N-GQD, (**b**) Bandgap of N-GQDs.

The electron density and electrostatic potential distributions of N-GQDs are shown in Figure 7. In GQDs, the carbon framework indicates relatively low electrostatic potential, while the edge regions exhibit higher electrostatic potential. Additionally, the electron cloud distribution within the hexagonal rings of GQDs demonstrates a certain degree of symmetry. For G-NG and P-NG (Figure 7a_2_,a_3_), the electrostatic potential increases in the nitrogen-doped regions. Pyrrolic nitrogen at the edge exhibits a higher electrostatic potential, indicating a stronger electrophilic attack capability. This can make pyrrolic nitrogen a potential active site for pseudocapacitive reactions. Moreover, the local electrostatic potential differences induced by nitrogen doping may drive electrons to transfer from low-potential regions (blue) to high-potential regions (red). This charge redistribution enhances the conductivity and electrochemical capability of N-GQDs. For GP-NG (Figure 7a_4_), the electrostatic potential around graphitic and pyrrolic nitrogen slightly increases, which is likely attributed to enhanced local reactivity resulting from the electron-donating effect of C-N bonds introduced by nitrogen doping. This also indirectly confirms the synergistic interaction between graphitic and pyrrolic nitrogen, where pyrrolic nitrogen transfers electrons to the π-conjugated system through resonance effects, thereby improving the conductivity and activity of N-GQDs. To gain further insight into the role of graphitic and pyrrolic nitrogen in modulating the reactivity of N-GQDs, the HOMO and LUMO charge distributions were examined, as shown in Figure S12. In the case of undoped GQDs, these orbitals are primarily distributed along the edges. In G-NG, the charge distributions of HOMO and LUMO are primarily localized around graphitic nitrogen, while in P-NG, the HOMO electron density is higher near pyrrolic nitrogen, indicating the strong electron-donating capability of pyrrolic nitrogen, which makes it an active center for electrode reactions. The bandgap (E_g_) of N-GQDs, representing the energy required for electrons to transition from HOMO to LUMO, is calculated (Figure 7b). The bandgap of GQDs is 1.92 eV, while the bandgaps of G-NG and P-NG are significantly reduced to 0.28 eV and 0.21 eV, respectively, indicating that nitrogen doping can drastically lower the energy required for electron transitions, facilitating electron transfer within the material. For GP-NG, the bandgap is further reduced to 0.19 eV, suggesting higher redox reactivity. This low bandgap once again validates the synergistic effect of graphitic and pyrrolic nitrogen.

**Figure 8:**
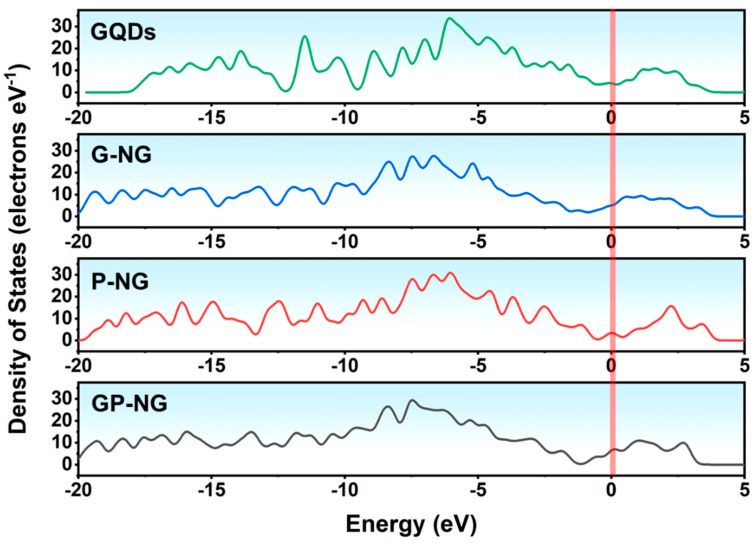
The density of states of N-GQDs.

Figure 8 shows the total density of states (DOS) curves for N-GQDs. The DOS near the Fermi level (0 eV) for all N-GQDs is greater than zero, indicating good conductivity. Compared to P-NG, G-NG exhibits slightly higher DOS, likely due to the stronger coupling ability of graphitic nitrogen with the π-conjugated system and its greater contribution to electronic states near the Fermi level. Notably, GP-NG shows the highest DOS, demonstrating superior electron transport capability and electrochemical activity.

The superior electrochemical performance of GP-NG originates from: (1) Synergistic graphitic–pyrrolic nitrogen interactions, in which pyrrolic N facilitates charge transfer and graphitic N reinforces electronic coupling. (2) Reduced bandgap and high density of states near the Fermi level can facilitate electron transitions and improve reactivity in redox reactions. (3) Optimized charge redistribution enhances conductivity and the activation of nitrogen-doped regions. These factors collectively enable GP-NG to exhibit exceptional conductivity, pseudo-capacitance, and electrochemical stability, highlighting its great potential for high-performance energy storage systems.

## 4. Conclusions

In this paper, N-GQDs with controllable size and structure were synthesized by a facile hydrothermal method. The reaction parameters significantly affected their morphology and electrochemical properties. Under optimal conditions (180 °C, 6 h), NG-2 exhibited a uniform size of 3.42 nm, abundant functional groups, and suitable structural defects, providing rich active sites. The NG-2 electrode showed a high capacitance of 309.8 F g^−1^ at 1 A g^−1^ with 98.1% retention after 8000 cycles. In addition, the NG-2//AC-ASC device delivered 22.5 Wh kg^−1^ at 500 W kg^−1^ and retained 85.7% capacitance after 8000 cycles, confirming its excellent performance and application potential. The synergistic mechanism of graphite nitrogen and pyrrole nitrogen in improving the electronic structure and surface activity of N-GQDs was proposed by comparing the electronic structures of N-GQDs with different nitrogen doping types. Combined with the electrochemical properties of N-GQDs, the energy storage mechanism of N-GQDs was elucidated, providing theoretical support for their application in SCs.

## Data Availability

The original contributions presented in this study are included in the article and Supplementary Materials. Further inquiries can be directed to the corresponding authors.

## Supplementary Materials

The following supporting information can be downloaded at: https://www.mdpi.com/article/10.3390/ma18235373/s1, Figure S1: (a) Optical photograph of N-GQDs solution; (b_1_,b_2_) Optical photos of NG-2 under fluorescent lamp and 365nm ultraviolet lamp, respectively; Figure S2: Height distribution of N-GQDs; Figure S3: Particle size distribution of NG-2; Figure S4: (a) 3D image; (b) AFM image, the illustration is the height distribution of NG-2; Figure S5: (a) Fitted curve of Z′ vs. ω^−1/2^ of, (b) C″ (ω) vs. *f* (Hz) curve of NG electrode; Figure S6: The CV curve:(a) NG-1, (a) NG-1, (b) NG-3, (c) NG-4, (d) NG-5, (e) NG-6; Figure S7: The b-value analysis of the N-GQDs electrode; Figure S8: Diffusion and capacitive contributions at different scan rates of NG-2:(a) 10 mV s^−1^, (b) 20 mV s^−1^, (c) 30 mV s^−1^, (d) 50 mV s^−1^, and (e) 100 mV s^−1^; Figure S9: The GCD curves: (a) NG-1, (b) NG-3, (c) NG-4, (d) NG-5, (e) NG-6; Figure S10: SEM image of the NG-2 electrode after 8000 GCD cycles; Figure S11: The model of N-GQDs: (a) GQDs, (b) G-NG, (c) P-NG, and (d) GP-NG; Figure S12: (a–d) The HOMO, (a_1_–d_1_) The LUMO of GQDs, G-NG, P-NG and GP-NG; Table S1: Hydrothermal synthesis parameters for different samples; Table S2: R_s_ and R_ct_ values of NG electrodes; Table S3: Comparison of the electrochemical performance of NG-2 with other GQDs-based electrode materials.
