# Supplementary material for: A Facile Synthesis Strategy for N-Doped Graphene Quantum Dots Electrode Materials: Electrochemical Behaviors and Universal Energy Storage Mechanism
Source: Materials (Basel). 2025 Nov 28;18(23):5373. doi: 10.3390/ma18235373 (PMC12693605; doi:10.3390/ma18235373)
Supplement: Supplementary file 1 [file materials-18-05373-s001.zip › materials-3932529-supplementary.pdf]

materials

Article

A Facile Synthesis Strategy for N-Doped Graphene Quantum Dots Electrode Materials: Electrochemical Behaviors and Universal Energy Storage Mechanism

Yongbo Wang [1,2,†], Shichao Dai [2,†], Jinghe Guo [2], Yanxiang Wang [2,*] and Bo Tang [1,2,*]

1 College of Chemistry, Chemical Engineering and Materials Science, Shandong Normal University, Jinan 250014, China; wyongbo1011@163.com
2 Key Laboratory for Liquid-Solid Structural Evolution and Processing of Materials, State Key Laboratory of Crystal Materials, Shandong University, Jinan 250061, China; 18264530353@163.com (S.D.); tongyjames@gmail.com (J.G.)
* Correspondence: wyx079@sdu.edu.cn (Y.W.); tangbo0765@163.com (B.T.)
† These authors contributed equally to this work.

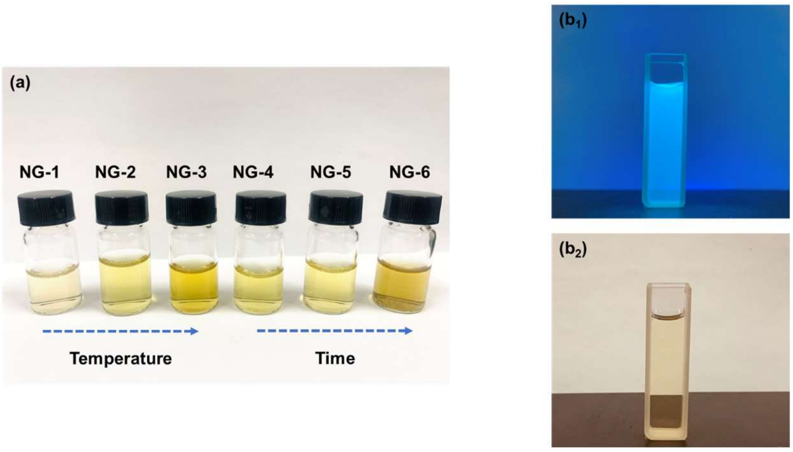

Figure S1. (a) Optical photograph of N-GQDs solution; (b_1-b_2) Optical photos of NG-2 under fluorescent lamp and 365nm ultraviolet lamp, respectively.

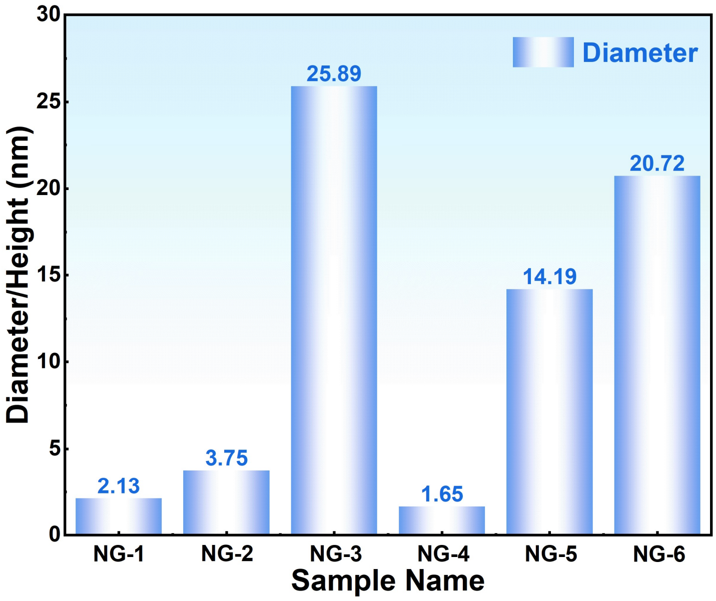

Figure S2. Height distribution of N-GQDs.

 https://doi.org/10.3390/10.3390/ma18235373

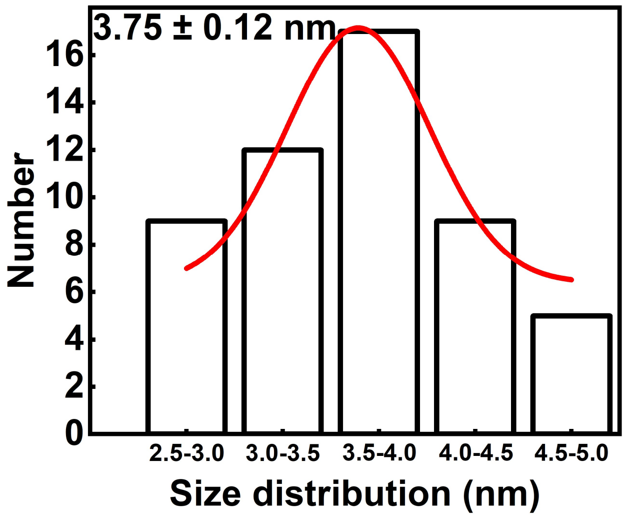

Figure S3. Particle size distribution of NG-2.

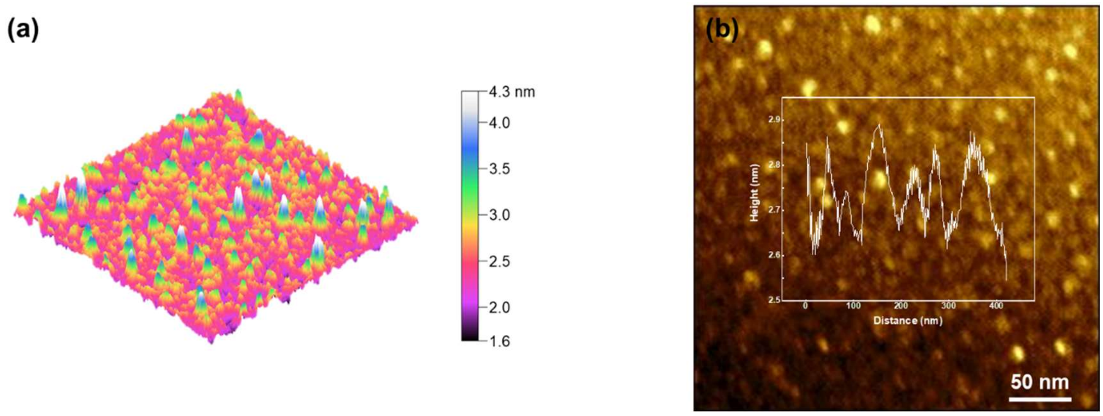

Figure S4. (a) 3D image; (b) AFM image, the illustration is the height distribution of NG-2.

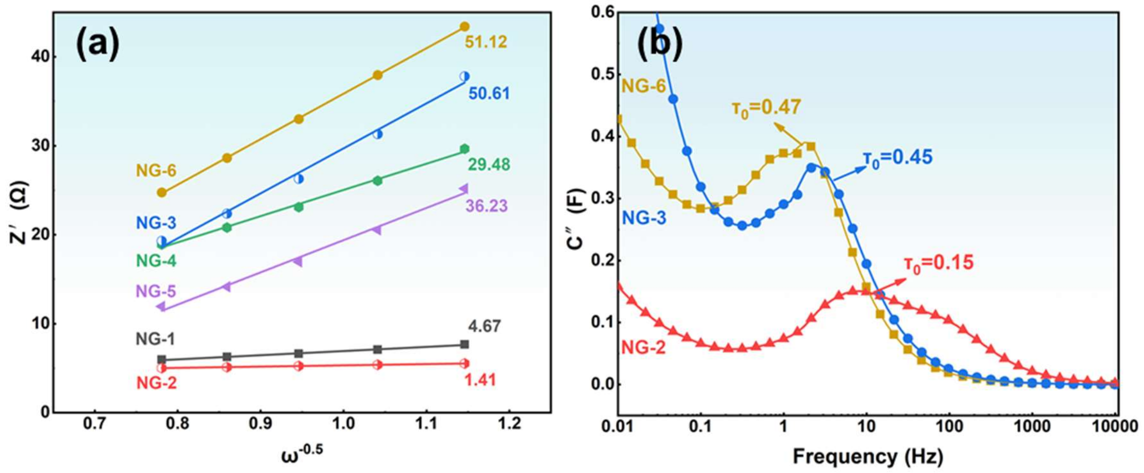

Figure S5. (a) Fitted curve of Z' vs. $\omega^{-1/2}$ of, (b) C'' (ω) vs. *f* (Hz) curve of NG electrode.

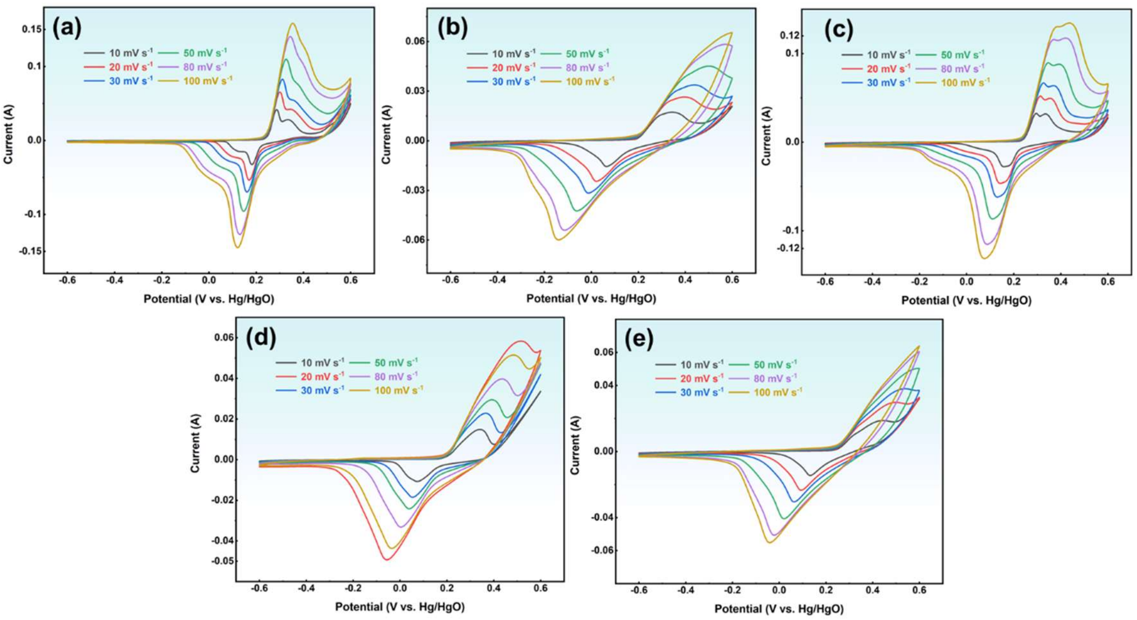

Figure S6. The CV curve:(a) NG-1, (a) NG-1, (b) NG-3, (c) NG-4, (d) NG-5, (e) NG-6.

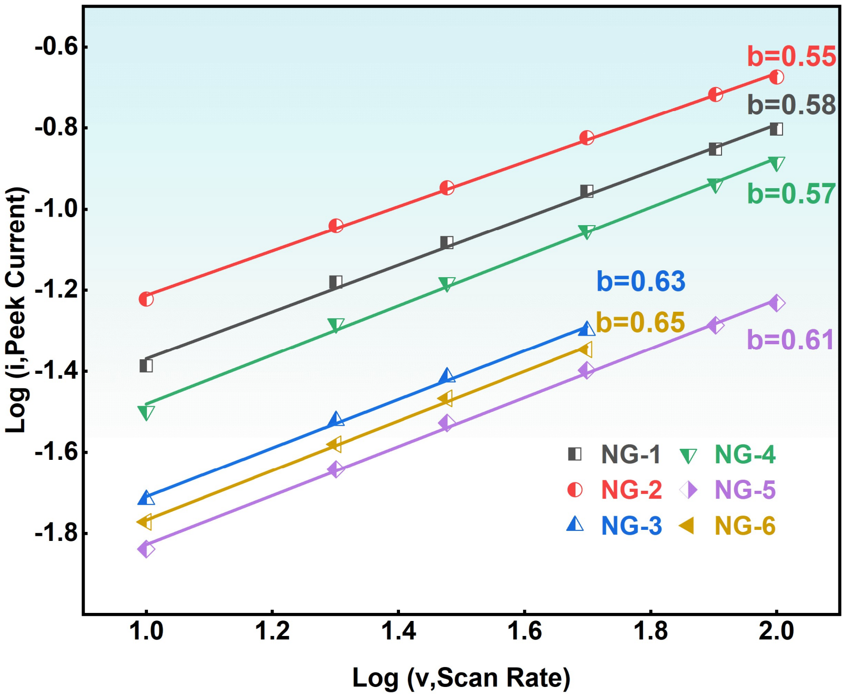

Figure S7. The b-value analysis of the N-GQDs electrode.

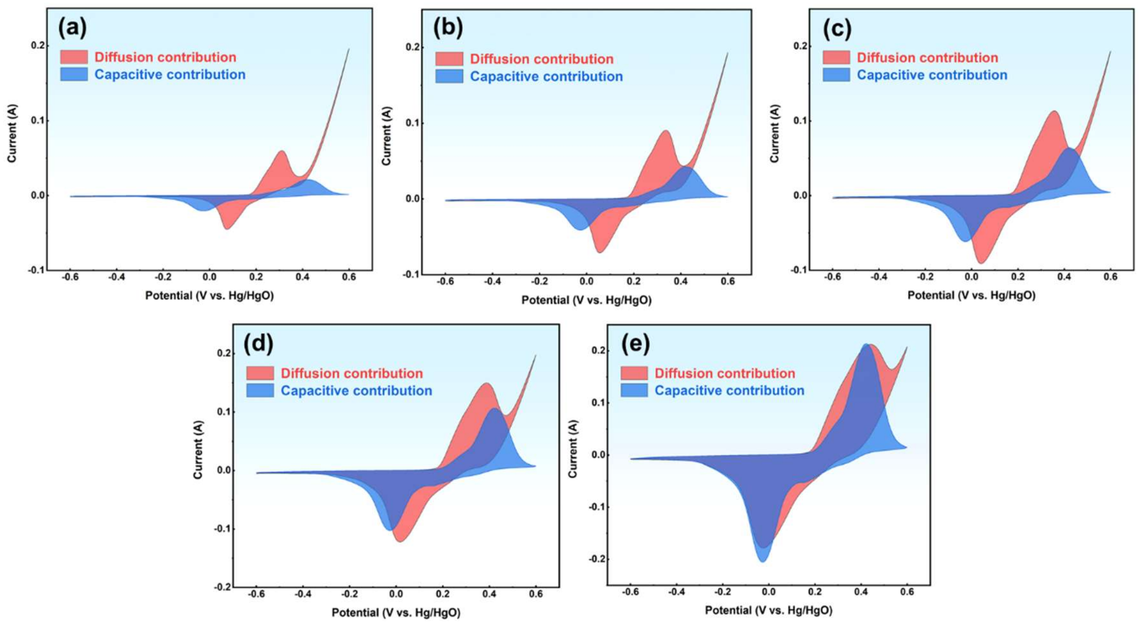

Figure S8. Diffusion and capacitive contributions at different scan rates of NG-2:(a) 10 mV s^{-1}, (b) 20 mV s^{-1}, (c) 30 mV s^{-1}, (d) 50 mV s^{-1}, and (e) 100 mV $s^{-1.}$

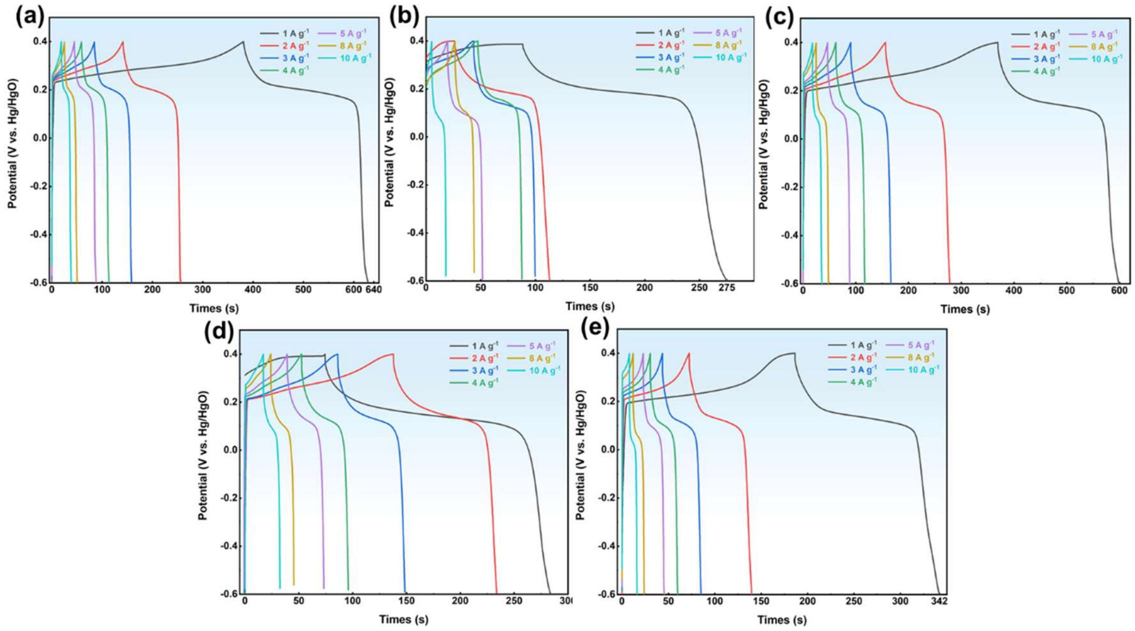

Figure S9. The GCD curves: (a) NG-1, (b) NG-3, (c) NG-4, (d) NG-5, (e) NG-6.

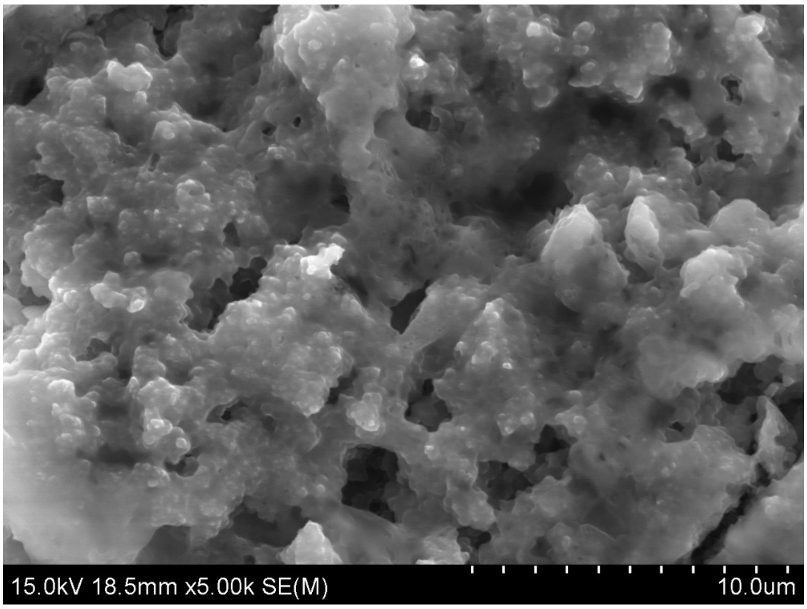

Figure S10. SEM image of the NG-2 electrode after 8000 GCD cycles.

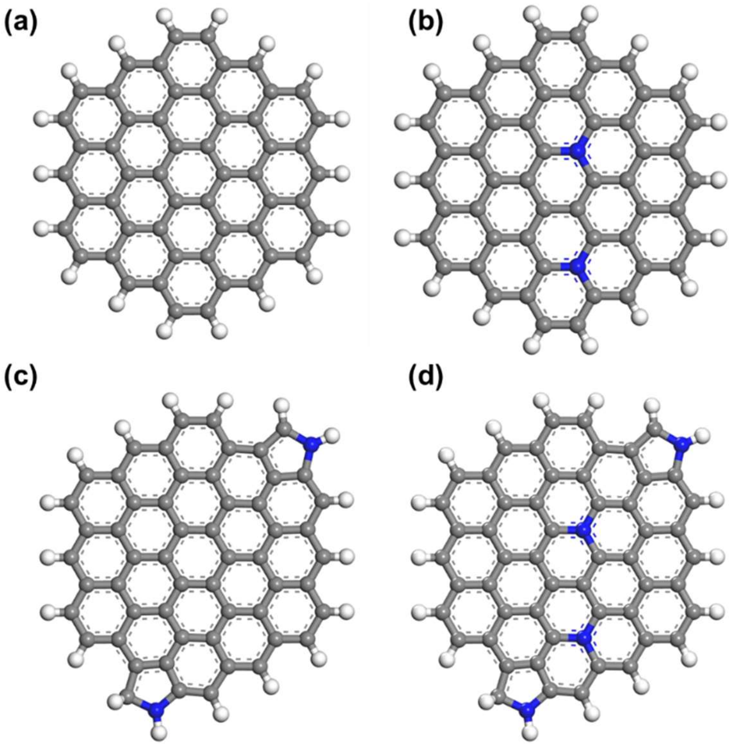

Figure S11. The model of N-GQDs: (a) GQDs, (b) G-NG, (c) P-NG, and (d) GP-NG.

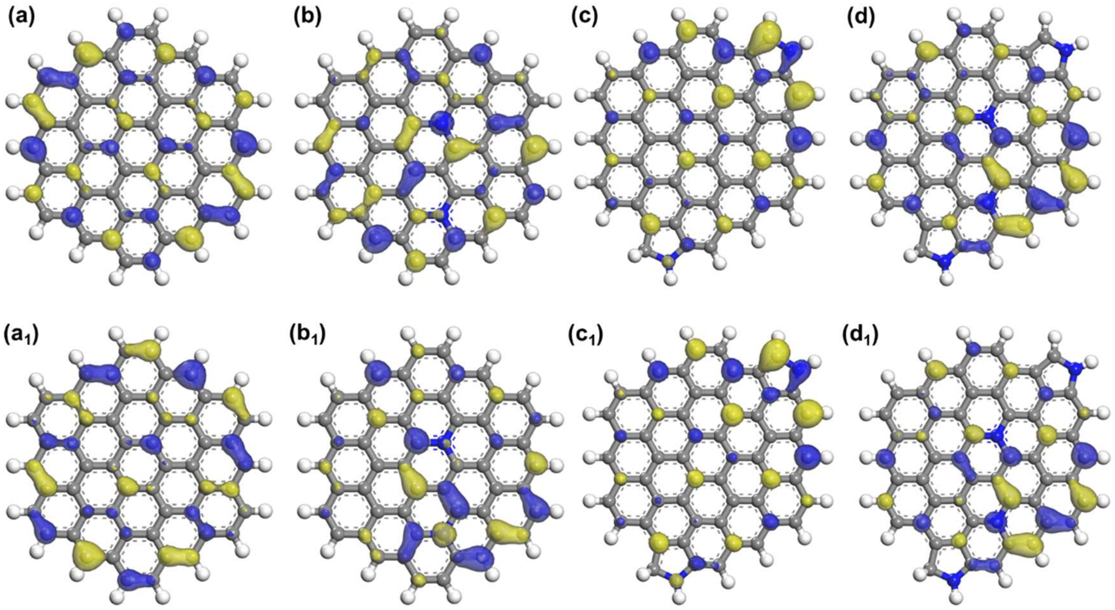

Figure S12. (a-d) The HOMO, (a_1-d_1) The LUMO of GQDs, G-NG, P-NG and GP-NG.

Table S1. Hydrothermal synthesis parameters for different samples.

Sample	NG-1	NG-2	NG-3	NG-4	NG-5	NG-6
Time (h)	6	6	6	4	8	12
Temperature (°C)	160	180	200	180	180	180

Table S2. R_s and R_{ct} values of NG electrodes.

Sample	R_{ct}	R_s
NG-1	/	2.76
NG-2	/	0.74
NG-3	/	4.12
NG-4	/	4.49
NG-5	/	4.21
NG-6	/	3.98

Table S3. Comparison of the electrochemical performance of NG-2 with other GQDs-based electrode materials.

Precursor	Product (size in nm)	Specific capacitance	Stability (cycles)	Energy density (Wh kg^{-1})	Power density (W kg^{1})	**Ref**
Citric acid	3.42 N-GQDs	309.8 F g^{-1} at 1 A g^{-1}	98.1 % (8000)	22.5	500	This work
Pyrene	A-GQDs	400-595 F g^{-1} at	90%	21.8	250	1

	(2.9±0.5)	1 A g^{-1}	(10 000)			
Pyrene	N, O-GQDs (4-10)	212 F g^{-1} at 0.07 A g^{-1}	89.7% (5000)	5.73	44.7	2
Pyrene	N-GQDs (0.5-5.5)	294 F g^{-1} at 0.5 A g^{-1}	94.1% (5000)	10.2	125	3
Pyrene	N-GQDs	40.4 F g^{-1} at 0.5 A g^{-1}	90.1% (5000)	14.4	401	3
1,3,6-trinitropyren	N-GQDs (0.5-5)	541 F g^{-1} at 0.5 A g^{-1}	90.9% (8000)	18.8	109	4
1,3,6-trinitropyren	N-GQDs (3.5)	400 F g^{-1} at 0.3 A g^{-1}	82% (5000)	14	89.5	4
ACS Material Company	GQDs (3.5)	291.86 F g^{-1}	98% (1000)	16.95	985	5
GQDs	GQDs (2-10)	315 F g^{-1} at 1 A g^{-1}	100% (10 000)	9.21	248	6

References

1. Li Z, Qin P, Wang L, Yang CS, Li YF, Chen ZW, Pan D, Wu M. Amine-enriched Graphene Quantum Dots for High-pseudocapacitance Supercapacitors. Electrochimica Acta. 2016;208:260-6.
2. Li Z, Li YF, Wang L, Cao L, Liu X, Chen ZW, Pan D Y, Wu M H. Assembling nitrogen and oxygen co-doped graphene quantum dots onto hierarchical carbon networks for all-solid-state flexible supercapacitors. Electrochimica Acta. 2017;235:561-9.
3. Li Z, Bu F, Wei JJ, Yao WW, Wang L, Chen Z, Pan D, Wu M. Boosting the energy storage densities of supercapacitors by incorporating N-doped graphene quantum dots into cubic porous carbon. Nanoscale. 2018;10(48):22871-83.
4. Li Z, Liu X, Wang L, Bu F, Wei JJ, Pan DY, Wu M. Hierarchical 3D All-Carbon Composite Structure Modified with N-Doped Graphene Quantum Dots for High-Performance Flexible Supercapacitors. Small. 2018;14(39).
5. Abidin S, Mamat S, Rasyid SA, Zainal Z, Sulaiman Y. Fabrication of poly(vinyl alcohol)-graphene quantum dots coated with poly(3,4-ethylenedioxythiophene) for supercapacitor. Journal of Polymer Science Part a-Polymer Chemistry. 2018;56(1):50-8.
6. Tian WH, Zhu JY, Dong Y, Zhao J, Li J, Guo NN, Lin H, Zhang S, Jia D. Micelle-induced assembly of graphene quantum dots into conductive porous carbon for high rate supercapacitor electrodes at high mass loadings. Carbon. 2020;161:89-96.
